# Supplementary material for: Infectiousness of Asymptomatic Meriones shawi, Reservoir Host of Leishmania major
Source: Pathogens. 2023 Apr 18;12(4):614. doi: 10.3390/pathogens12040614 (PMC10143307; doi:10.3390/pathogens12040614)
Supplement: Supplementary file 1 [file pathogens-12-00614-s001.zip › pathogens-2332866-supplementary.pdf]

Week p.i.	Meriones No.	Infective dose	Symptoms on the pinna	Xenodiagnosis (positive females/ dissected females)
4	1	7,0E+03	Asymptomatic	0/24
4	2	7,0E+03	Asymptomatic	0/19
4	3	7,0E+03	Asymptomatic	1/31
5	7	7,0E+03	Asymptomatic	0/20
5	8	7,0E+03	Asymptomatic	0/24
5	9	7,0E+03	Asymptomatic	0/33
5	10	7,0E+03	Asymptomatic	1/21
5	11	7,0E+03	Asymptomatic	0/19
5	12	7,0E+03	Asymptomatic	0/42
5	13	14,0E+3	Asymptomatic	0/17
5	14	14,0E+3	Asymptomatic	0/27
5	15	14,0E+3	Asymptomatic	0/14
5	18	14,0E+3	Asymptomatic	0/17
5	19	14,0E+3	Asymptomatic	0/19
5	20	14,0E+3	Asymptomatic	0/20
5	23	14,0E+3	Asymptomatic	0/16
5	24	14,0E+3	Asymptomatic	0/20
5	25	14,0E+3	Asymptomatic	0/18
5	28	14,0E+3	Asymptomatic	0/19
5	29	14,0E+3	Asymptomatic	0/20
5	30	14,0E+3	Asymptomatic	0/16
8	1	7,0E+03	Asymptomatic	1/27
8	2	7,0E+03	Asymptomatic	4/24
8	3	7,0E+03	Asymptomatic	1/15
10	7	7,0E+03	Asymptomatic	1/13
10	8	7,0E+03	Asymptomatic	0/10
10	9	7,0E+03	Asymptomatic	0/6
10	10	7,0E+03	Nodulus/swelling	0/18
10	11	7,0E+03	Asymptomatic	0/10
10	12	7,0E+03	Asymptomatic	0/20
10	13	14,0E+3	Asymptomatic	0/29
10	14	14,0E+3	Nodulus/swelling	2/10
10	15	14,0E+3	Asymptomatic	0/13
10	16	14,0E+3	Asymptomatic	0/22
10	17	14,0E+3	Asymptomatic	0/18
10	18	14,0E+3	Asymptomatic	0/21
10	19	14,0E+3	Asymptomatic	0/18
10	20	14,0E+3	Asymptomatic	1/17
10	21	14,0E+3	Asymptomatic	1/24

10	22	14,0E+3	Asymptomatic	0/24
10	23	14,0E+3	Asymptomatic	1/17
10	24	14,0E+3	Asymptomatic	0/16
10	25	14,0E+3	Asymptomatic	0/9
10	26	14,0E+3	Asymptomatic	0/24
10	27	14,0E+3	Asymptomatic	0/28
10	28	14,0E+3	Nodulus/swelling	3/25
10	29	14,0E+3	Asymptomatic	1/13
10	30	14,0E+3	Asymptomatic	0/28
10	31	14,0E+3	Ulcerative lesion	0/21
10	32	14,0E+3	Asymptomatic	2/24
12	1	7,0E+03	Asymptomatic	0/20
12	2	7,0E+03	Ulcerative lesion	2/22
12	3	7,0E+03	Ulcerative lesion	2/24
15	7	7,0E+03	Nodulus/swelling	0/13
15	8	7,0E+03	Ulcerative lesion	1/16
15	9	7,0E+03	Ulcerative lesion	0/23
15	10	7,0E+03	Nodulus/swelling	0/10
15	11	7,0E+03	Ulcerative lesion	1/10
15	12	7,0E+03	Ulcerative lesion	2/32
16	2	7,0E+03	Ulcerative lesion	1/6
16	4	7,0E+03	Ulcerative lesion	1/6
20	7	7,0E+03	Ulcerative lesion	0/17
20	8	7,0E+03	Ulcerative lesion	0/23
20	9	7,0E+03	Ulcerative lesion	0/30
20	10	7,0E+03	Nodulus/swelling	0/15
20	11	7,0E+03	Ulcerative lesion	0/29
20	13	14,0E+3	Ulcerative lesion	0/20
20	14	14,0E+3	Ulcerative lesion	1/7
20	16	14,0E+3	Nodulus/swelling	0/12
20	17	14,0E+3	Asymptomatic	0/18
20	18	14,0E+3	Asymptomatic	2/17
20	19	14,0E+3	Ulcerative lesion	0/19
20	20	14,0E+3	Nodulus/swelling	3/13
20	21	14,0E+3	Ulcerative lesion	0/14
20	24	14,0E+3	Ulcerative lesion	1/23
20	25	14,0E+3	Ulcerative lesion	0/22
20	26	14,0E+3	Ulcerative lesion	0/24
20	27	14,0E+3	Ulcerative lesion	0/14
20	28	14,0E+3	Asymptomatic	1/31
20	29	14,0E+3	Ulcerative lesion	2/20
20	30	14,0E+3	Ulcerative lesion	4/22
20	31	14,0E+3	Ulcerative lesion	0/20

20	32	14,0E+3	Ulcerative lesion	4/19
25	7	7,0E+03	Ulcerative lesion	1/25
25	8	7,0E+03	Ulcerative lesion	4/34
25	9	7,0E+03	Ulcerative lesion	0/36
25	10	7,0E+03	Nodulus/swelling	1/33
25	11	7,0E+03	Ulcerative lesion	1/32
25	16	14,0E+3	Ulcerative lesion	1/3
25	20	14,0E+3	Ulcerative lesion	1/2
25	24	14,0E+3	Ulcerative lesion	1/3
25	30	14,0E+3	Ulcerative lesion	1/1
27	3	14,0E+3	Asymptomatic	1/22
30	7	7,0E+03	Ulcerative lesion	0/28
30	11	7,0E+03	Ulcerative lesion	0/36
30	13	14,0E+3	Ulcerative lesion	0/12
30	14	14,0E+3	Ulcerative lesion	1/12
30	15	14,0E+3	Ulcerative lesion	0/14
30	16	14,0E+3	Ulcerative lesion	0/12
30	17	14,0E+3	Nodulus/swelling	0/16
30	18	14,0E+3	Ulcerative lesion	2/12
30	19	14,0E+3	Ulcerative lesion	0/15
30	20	14,0E+3	Ulcerative lesion	0/12
30	24	14,0E+3	Ulcerative lesion	0/20
30	25	14,0E+3	Ulcerative lesion	0/21
30	26	14,0E+3	Ulcerative lesion	0/18
30	27	14,0E+3	Ulcerative lesion	0/20
30	28	14,0E+3	Asymptomatic	1/20
30	29	14,0E+3	Nodulus/swelling	1/21
30	30	14,0E+3	Ulcerative lesion	0/17
30	31	14,0E+3	Nodulus/swelling	0/15
30	3	14,0E+3	Asymptomatic	1/16
38	3	14,0E+3	Asymptomatic	0/30

Table S1. Results of xenodiagnostic experiments performed with *Phlebotomus papatasi* on *Meriones shawi* infected with *Leishmania major*.
